# Incorporating clickers into an enzymology course improves student performance

**DOI:** 10.1002/2211-5463.12599

**Published:** 2019-02-18

**Authors:** Claire Stines‐Chaumeil, Patrick Paumard, Mark A. Hooks

**Affiliations:** ^1^ CNRS Univ. Bordeaux CRPP, UMR5031 Pessac France; ^2^ UMR 5095 CNRS IBGC Bordeaux France; ^3^ Laboratory of Membrane Biogenesis University of Bordeaux/CNRS Villenave d'Ornon France

**Keywords:** active learning, audience response system, clickers, enzymology courses, tutorials

## Abstract

Here, we describe how poor exam results of undergraduate students enrolled in an enzymology course at the University of Bordeaux were improved through the introduction of ‘clickers’ as an audience response system. By using clickers only in a small‐group tutorial element of a large theoretical course, we observed an improvement in exam scores that resulted in a lower failure rate for the course. Furthermore, students of all abilities were found to benefit from their use. Students reported better retention of both lecture and tutorial content. An analysis of how clickers were employed within the tutorials indicated that the use of clickers to promote discussion and impart knowledge likely resulted in a moderate improvement of exam scores. We hypothesize that students were more prepared for exams through greater reflection of exam questions, resulting in an enhanced ability to retrieve memorized information and apply it within a time‐limited exam setting.

**Abbreviations**
ARS: audience response system
MCQ: multiple choice question

Audience response systems (ARS) or ‘clickers’ are pedagogic tools that survey the level of knowledge of students and provide feedback to both the students and instructor during the teaching session 1. This concept was popularized by the physicist Eric Mazur of Harvard University, who implemented peer‐learning quizzes during his classes 2, 3, whereby students were encouraged to interact amongst themselves to arrive at the correct answers 4. As has been shown for a number of other disciplines 5, clickers create an environment where students work with and learn from each other 6. This interaction is the basis of ‘active learning’ that seeks to promote collaborative work among learners 7, which is regarded as essential for the learning process. Freeman *et al*. 8 employed clickers between 10% and 15% of the teaching time within the framework of small classes in science, technology, engineering and mathematics. They arrived at the conclusion that there is a direct relationship between clicker use and active learning 8, which has been supported by another study linking clicker use to increased group dynamism among biology students 3.

This study presents the data obtained from a population of general biology students taking the Enzymology course during their second year of a 3‐year degree program. Teaching enzymology is challenging to biology students as they perceive this subject to be difficult to grasp due to the use of mathematical equations, such as hyperbolas and exponentials. Students were considered to be underperforming consistently in the Enzymology course compared to other biology courses. We postulated that introducing clickers as an ARS would motivate students to learn and engage with the course content and ultimately would improve student performance in exams. The study was initiated with three general objectives: (a) to improve teaching practices through the use of novel and sustainable teaching technologies, (b) to place the student at the center of his/her learning by developing further interaction between students and their teachers and peers, and (c) to enhance the intrinsic motivation of students and to stimulate group discussion.

For this study, clickers were employed in small‐group tutorial sessions where the theoretical content on enzymology had been delivered to larger groups within a lecture theatre environment without the use of clickers. Within the tutorial setting, clickers were used at key moments to maintain students’ attention, to evaluate anonymously the acquisition of knowledge from the lectures, and to permit anonymous student participation while encouraging collaborative work. Foreseeable advantages were providing students with immediate feedback on their level of understanding and to allow instructors to adapt on the moment their teaching content to increase understanding. Subsequently, clicker data would guide individual student study of course material and help instructors to modify course content where necessary. The success of the study was evaluated quantitatively as a significant improvement in exam scores and lower failure rates and qualitatively as the satisfaction of students with the use of clickers as a teaching tool.

## Experimental design

### Ethical considerations of the study

The project was undertaken with the approval of the ethics committee for teaching affairs of the University of Bordeaux. Students were informed prior to the start of instruction of the purpose and objectives of the investigation. Student participation was anonymous, and each student was presented with the opportunity to exclude him/herself from the study at any time. Furthermore, students were assured that participation would have no bearing on any score assignment and that the results could be used for publication.

### Context and content of the Enzymology course

All students encompassed within the study were enrolled in the Biology Department at the University of Bordeaux. The Enzymology course as the focus of the study occurred in the second year of a 3‐year degree program between the years 2013 and 2015. Enzymology forms one‐half of the biochemistry teaching of the second year along with Metabolic Biochemistry, which was not included in the study. Enzymology/Metabolic Biochemistry ran concurrently with five other biology courses: Cell Biology, Genetics, Plant Physiology, Thermodynamics, and Cellular Physiology.

The 3‐year study was divided up with years 2013 and 2014 serving as controls in which students were taught according to established methods, and clickers were introduced in 2015. The same course content, exercises, handouts, slides, instructors and assessments were used during the 3‐year study. The numbers of students in the class over the three study years were 124, 143 and 168, respectively. Each year of the study, the Enzymology course was organized around three delivery approaches: (a) lectures with theoretical content, (b) hands‐on practical experience in problem solving and (c) tutorials for demonstrating standard mathematical equations, such as the Michaelis–Menten equation, accompanied by exercises. Theoretical content was delivered in three, 2‐h sessions (once every other week) by one instructor in a large audience setting of all students. Lectures were supplemented by tutorials, i.e. three tutorial sessions in total, where students were divided into small groups of 20–30 per group. Groupings had been organized outside the course by the Department as a function of time scheduling and did not reflect any past course performance. The hands‐on practical component entailed three 4‐h sessions of direct experimental measurement and data manipulation in groups of about 16 students and directed by one instructor. The use of clickers was introduced during tutorials, where the students were divided into six groups with 20–30 students per group overseen by one instructor. Clickers were used by all groups. Furthermore, teaching was delivered in an interactive manner where students were allowed to discuss and correct the exercises after revelation of the initial responses.

The primary topic in Enzymology was the study of the kinetics of a one‐substrate Michaelian enzyme with and without a non‐allosteric inhibitor. The topic was divided into five parts. The first part defined an enzyme, its catalytic specificity, and the concept of an active site covering binding and catalysis. The second part introduced the Michaelis–Menten equation, and the third part covered the effects of rapid and reversible inhibitors on a non‐allosteric enzyme. The fourth and fifth parts, respectively, discussed the effect of reaction environment, such as temperature and pH, on enzyme activity and protein–ligand interactions and the techniques for analyzing binding equilibrium, such as dialysis at equilibrium and Scatchard diagrams.

### Application of clicker technology within the teaching setting

The clickers chosen for the study were purchased from Turning Technologies (www.turningtechnologies.com). In addition, turningpoint ^®^ software (Turning Technologies) was linked to microsoft powerpoint ^®^ to create slides for immediate class viewing of questions and output. Clickers were distributed to the students at the beginning of each tutorial session and recovered at the end. Student participation was anonymous and non‐gradable, as student identity was not associated with a clicker serial number and clickers were distributed randomly for each session. During the tutorial session, multiple‐choice or True/False questions were presented at various times and students were asked to respond with the answer that they deemed correct. The number of possible responses of each clicker was tuned to the type of question – two choices for a True/False question, for example – and responses were received by a key receptor connected to a computer with video projector. The results as a percentage of each possible answer for each question were shown. Tutorial sessions progressed according to the percentage of correct answers provided. With ≥ 70% correct answers, the tutorial session continued unabated. From 30% to 69% correct answers, the students were given the choice of a revote with 1 min of discussion among themselves. Depending on the outcome of the revote, the students were invited to continue the tutorial or the instructor further explained the solution. In order to avoid any bias for the revote, students were not permitted to know individual votes until the end of the voting session for a particular question 9. For a response of < 30%, the instructor would intervene and further explain the concept of the question and provide the correct answer.

### Types of tutorial questions

The question bank comprised 28 elements of which the vast majority (25) represented knowledge retention of both lecture and tutorial material. The other three questions were introductory to the tutorial sessions, such as ‘Before today's tutorial, had you ever heard about Scatchard?’. The 25 knowledge‐retention questions demanded either one or more correct answers from multiple choice questions (MCQ) or a True/False response. At least seven questions were asked in each of the three tutorial sessions for each of the six tutorial groups for a total of 168 questions presented during the course. Over the duration of the course, each student experienced 28 questions.

### Student evaluation on the use of clickers

Student evaluation was obtain through a written survey of eight questions (yes/no, MCQ, and Likert scale) aimed at registering the experience and feelings of students regarding the use of clickers. The questionnaire also allowed students to provide comments. The surveys were made available to students through a Web‐based interface of the University Virtual Learning Environment. Students’ participation in the survey was voluntary and anonymous. This feedback was collected at the end of the course, but before publication of final course results. The questionnaire contained the following elements: (a) Did you find it easy to use the clickers? (b) Was the content of the course more understandable? (c) Were the tutorials more interactive? (d) Did you gain time during the preparation of exams due to the questions asked with clickers? (e) Have you ever used clickers before? (f) Would you like to use clickers in other courses? (g) Would you also like to use clickers in lectures? (h) What pedagogic support would you like to have for Enzymology courses?

### Assessing student success and analysis of test results and failure rates

A non‐mandatory pre‐test of 10 questions was implemented on an online platform before the first Enzymology lecture. The aim was to evaluate student knowledge of kinetics prior to the onset of teaching. This pre‐test was based on the classic chemical kinetics studied in the previous semester, in that chemical kinetics served as the theoretical basis for introducing and explaining enzyme kinetics. Student understanding of the course content was assessed in two ways, firstly through a midterm assignment that only included tutorial content and secondly through a final examination incorporating both lecture and tutorials content. For statistical analysis, the data from the two exams were treated independently as explained in Results. Statistical significance testing of exam scores between years was done in r (version 3.5.1) 10 using the resident statistical package. Plots were done in r using ggplot2 11. For failure rates, statistical significance was determined by one‐factor ANOVA using qtiplot statistical software (https://www.qtiplot.com).

## Results

The aim of this study was to determine if the use of an ARS would improve student performance in enzymology, which is considered by students to be a difficult and challenging subject to master. An online test was administered at the beginning of the course with the objective of monitoring student retention of information from previous, similar courses, and to habituate them from the outset to spot tests. The online pre‐test was administered only in 2015, the year that clickers were introduced. Of all the students enrolled, 58% of students responded to the online pre‐test. The mean score was of 73.5 ± 15 and the median score was 72.5 with respectively the 25th and 75th percentiles of 63.7 and 82.5.

### Clickers represented a novel learning technique and were well received by students

**Figure 1:**
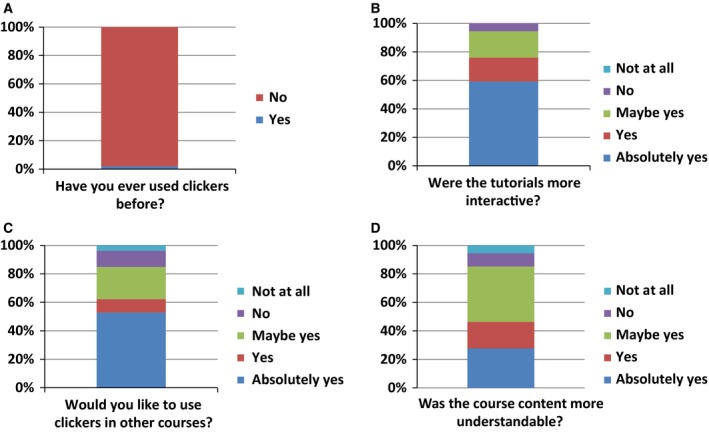
Student sentiment toward the use of an ARS. The questions posed to the students are given in ‘Experimental design’. For question A, the proportion of ‘No’ and ‘Yes’ respondents is shown. For questions B, C and D, the formulation of responses was based on the Likert scale 25, and the percentages reflect the proportion of students choosing a particular category.

Students’ opinions of the ARS were collected in year 2015 using an electronic survey hosted by the University's web service. Of the 168 students registered in 2015, 36% participated in the survey. The novelty of the ARS system was apparent in that 96% of respondents had never experienced clicker technology within a teaching context (Fig. 1A), and 100% of respondents found the clicker technology easy to use. Respondents were almost unanimous that the tutorial experience was more interactive with 95% agreeing with this position (Fig. 1B), and more than 80% would like to have clickers introduced into the lecture component and other courses (Fig. 1C). Importantly, 80% of all respondents signaled that they understood better the course content as a result of the ARS (Fig. 1D).

### Exam scores improved and failure rates decreased in the year with ARS

**Figure 2:**
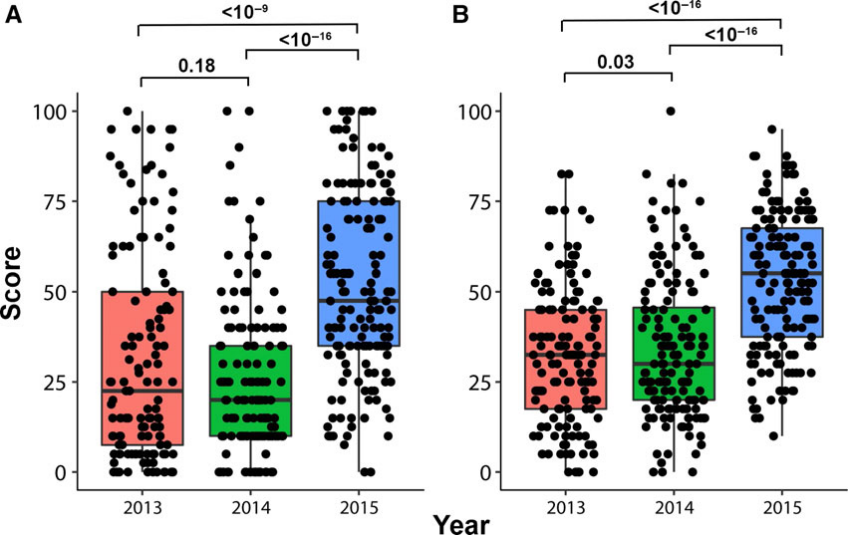
Comparison of exam scores without and with ARS. Boxplots showing the medians and ranges of the scores for the midterm assignment (A) and the final exams (B) for the three years 2013–2015. The clickers were used in 2015. The boxes provide the median score as denoted by the gray horizontal lines, and the lower and upper hinges represent the 25th and 75th percentiles, respectively. The whiskers represent plus and minus 1.5 times the interquartile range. The bars above the boxes relate to the direct comparison made between years, and the values represent the significance (*P*) according to a Wilcoxon rank sum test. The black circles represent the actual scores, which were 123, 146 and 163 for the midterm assignment and 139, 152 and 169 for the final exam for the years 2013–2015, respectively. The fewer scores than students for each year reflected some students missing an exam.

Any indication that students’ results were better in 2015 compared to the previous two years would indicate that the ARS helped students with the course. We first determined that the two control years of 2013 and 2014 would serve as a good baseline for comparison with 2015, because there was no difference in exam results between these two years (Fig. 2A,B). For the control years, the median of the scores for the midterm assignment and the final exam was just under and over 25%, respectively. Upon introduction of clickers in 2015, the median of the scores increased to around 50%. The 50% mark represented the cut‐off point between passing and failing the course, and thus, it represented a major performance target for the students. Because the final score for any student comprised both exam components, it was not immediately clear from these results how the student cohort, as a whole, had performed regarding this performance target. Therefore, the failure rates were calculated for each exam and the overall failure rate for the course as the percentage of students scoring below 50% (Table 1). The failure rates for both exams in 2015 decreased substantially, and these results translated into an overall failure rate for 2015 that was 1.9‐fold and 1.6‐fold less than observed for 2014 and 2013, respectively. These results for both the exam scores and the failure rates between control and test years were highly statistically significant. It should be noted that it is assumed that the performance potential among the yearly cohort of students was similar. This would represent the only potentially confounding factor, since staff, materials and course organization were identical over 3 years.

**Table 1:** Comparison of failure rates before and after introduction of the ARS. The failure rate was the percentage of students scoring < 50%. The failure rate for the exams was calculated based on all scores collected. The overall failure was determined from only those students taking both exams. Values are the percentage of students scoring < 50% on each of the exams. By ANOVA, *P* values for the midterm evaluation were *P* = 1.72 × 10^−20^ between years 2014 and 2015 and *P* = 1.08 × 10^−12^ between years 2013 and 2015; for the final exam *P* = 2.81 × 10^−17^ between years 2014 and 2015 and *P* = 7.57 × 10^−20^ between years 2013 and 2015

Year	Clicker	Failure rate		
		Midterm	Final	Overall
2013	No	73.1	81.2	77.0
2014	No	86.3	76.3	88.1
2015	Yes	51.2	39.2	42.3

**Figure 3:**
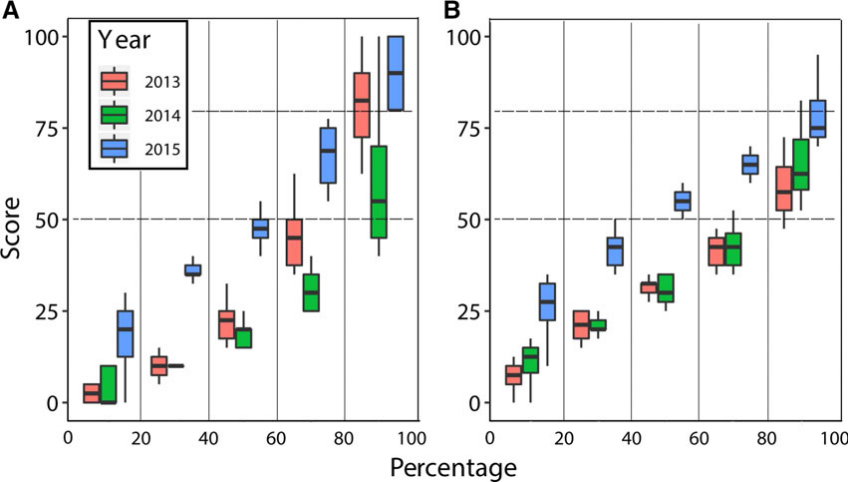
Comparison of scores across the performance scale. Boxplots showing the medians and ranges of the scores for the midterm assignment (A) and the final exams (B) for the three years 2013–2015. For each year, the cohort of scores was divided into five ranges of percentage as indicated by the solid vertical lines and these scores were plotted together. The statistics presented by the boxplots are given in the legend to Fig. 1. The individual scores were omitted to simplify the plots. The lower (50%) and upper (80%) horizontal dashed lines represent the pass and honorable‐mention points, respectively.

There are two key success levels in the University of Bordeaux marking and evaluation system, which are nearly universal within France and territories and countries that have adopted a French‐based education system. Any mark below 50% is considered as a fail and any mark above 80% receives an honorable mention (Fig. 3, dashed lines). Because there are defined success levels, it was informative to determine if there was a benefit to students across the performance spectrum, in addition to decreasing the failure rate. If we assume that students from 2015 were inherently equal in ability to those from 2013 to 2014 – which was supported by similar ranges of exam scores in other courses – we can expect that there would be a range of performance from very poor (scores < 20%) to very good students (scores > 80%). For the midterm assignments and final exams for the three years, we calculated the number of students falling within each of five percentage ranges and compared them among years (Fig. 3). For both the midterm assignment (Fig. 3A) and final exam (Fig. 3B), each range from 0% to 80% exhibited an increase in median score by about 20% in year 2015. This suggested that students of all abilities benefited almost equally from the ARS. Enhancements in median scores for the ranges 80–100% were also observed, but they were modest. An increase of 20% was observed only for the midterm assignment between years 2014 and 2015. A limited benefit to the best performing students was expected in that no score above 100% was achievable. It also seems that the cohort of students potentially gaining honorable mention (independent of ARS) varies from year to year compared to other percentage cohorts. Therefore, the benefit to this cohort of students would be less difficult to predict from year to year.

### Peer discussion and revote was not a major aspect of ARS use

**Figure 4:**
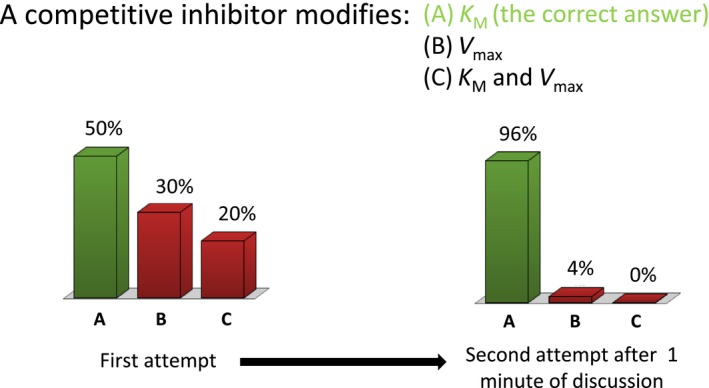
Example of score improvement upon peer discussion. The panel provides the enzymology‐related question, the choices of answer available, and the distribution of answers chosen before (left) and after (right) peer discussion and vote by ARS. Student participation in the use of clickers was 100%. The correct answers are shown in green and the incorrect answers are shown in red.

As mentioned previously, clickers as the ARS were introduced only into the tutorial component of the teaching program. An example of a question posed to the students that would form the basis of an ARS response is given in Fig. 4. This question was representative of required knowledge acquisition toward an understanding of enzyme kinetics, and it was demonstrative of the positive effects of student interaction. The outcome of this question revealed that initial presentation of the question led to as many as 50% of the students registering the wrong answer. Following instruction criteria, which dictated that if the percentage of correct answers was from 30% to 69%, the students were given 1 min to discuss the result prior to the revote. In this case, the revote showed that < 4% of students held to an incorrect answer. Noting the few choices of answers allowed, the fact that the technology was thoroughly explained to the students, and that students deemed clickers to be easy to use, it was evident that percentages adequately reflected student knowledge, and percentages were unlikely influenced by erroneous button pressing. However, out of the 168 total questions presented to the various groups throughout the course, on only 14 occasions (< 10%) did a question fall within the range where a revote was solicited or it was deemed necessary by the students to hold a revote. This demonstrated that discussion and revoting were not a major knowledge transfer mechanism. Students already possessed the required knowledge, or at most, needed modest intervention from peers or instructor to arrive at the correct answer.

## Discussion

In France 12, as in other parts of the world 8, University attendance is increasing dramatically, university enrollment has become more global, and the social backgrounds and aptitudes of applicants are more diverse. Innovation towards teaching large, diverse student populations has become essential in order to ensure maximum completion rates. One such teaching innovation is the use of clickers, which permit more interaction between instructors and students 13. Clickers as an ARS have been used in various educational contexts throughout the world for such disciplines as Veterinary Medicine, Physics, Mathematics, and Dentistry 14, 15, 16, 17. To our knowledge, this is the first time that clickers have been used in an enzymology‐related course. Enzymology is a fundamental topic in a number of scientific disciplines, such as biology, biological chemistry and biochemistry, biotechnology, and protein crystallography. It must be accepted that biology students deem enzymology to be a difficult topic. It is the responsibility of instructors to find ways of increasing interest and educational performance at the university undergraduate level in order to attract future students into the field. This study explored the use of clickers as an ARS to increase enthusiasm and knowledge in enzymology and to improve exam results.

Clickers have been used primarily in large‐group, lecture settings where up to several hundred students may be present in the same lecture hall. In fact, 80% of all ARS studies have been conducted using larger lecture‐type classes 18, and only a few papers have discussed the influence of group size on ARS effectiveness 19, 20, 21, 22. Our results have in common with these reports the observation that clickers are perceived well by the students and that they enhance participation and peer discussion. These reports also tried to distinguish the influence of clickers from that of general discussion, but we operated from the point of view that clickers comprise a tool to promote discussion. In France, the theoretical content of a topic is presented to all students simultaneously within a large lecture hall, but it is supported by tutorial sessions in which the large student cohort has been divided into several small groups for more detailed discussion. We decided to use clickers only during the tutorial sessions while maintaining the large‐group lecture format. To our knowledge, this is the only study conducted, thus far, where clickers were employed only during tutorial sessions. Students’ sentiment on the use of clickers was supportive as they found instruction more interactive and the theory content of the course more comprehensible. These results agree with those from other studies that have observed mostly positive attitudes towards clicker use 6, 22, 23. Furthermore, we observed that the incorporation of clickers into our Enzymology course improved exam scores and decreased failure rates. We found that this to be as effective as using them in a large group when evaluated against reports for other disciplines that have employed them in the more traditional large classroom environment. We observed as well an increase in student engagement, enhanced motivation and participation, and greater satisfaction with the course 14, 15, 16, 17.

It is important to reflect on how use of the ARS led to the improvement in student performance in our study. Certainly, the tutorial quizzes allowed the instructor to evaluate immediately student knowledge in order to cover concepts that were not clear, thus improving student understanding. Another possibility was that students gained knowledge of enzymology theory from the tutorial questions that could be applied directly to answer similar exam questions. In this respect, the peer discussion and revote of tutorial questions would have helped by providing students with answers. However, the extent to which exam scores were improved in 2015 suggests a limited influence from these two factors. Less than 10% of questions were open to peer discussion or it was deemed unnecessary to revote to correct student understanding. This equated to only two questions per student to which a direct answer to an exam question could be obtained and so this could not explain the 20% improvement. We note here that the exams were constructed so as not to repeat tutorial questions. Furthermore, peer discussion and supplemental instructor explanation would likely have less of a positive effect on the best performing students (range 80–100%), who clearly exhibited improved exam scores as well. The most likely explanation was that the tutorial exercises prepared students to better understand exam questions. Repeated use of the clicker technology likely improved the ability of students to analyze questions and to arrive at more correct answers within the restricted time frame of the exam. Within this context, the pre‐test would have served the same purpose. Students would have been prepared from the outset to recognize and interpret questions formulated by a set group of instructors and to retrieve from memory and apply existing knowledge. Furthermore, feedback from the students about the pre‐test indicated that instruction began with a positive outlook.

### Perspectives for the employment of ARS and future studies

There are a number of points from this study that require further reflection: (a) did the pre‐test have any influence on course performance? (b) Would an increase peer discussion time and revote have a corresponding larger influence on exam scores? (c) Would it be possible to evaluate the benefit of ARS use for the best performing students by including a sliding upper scale? (d) Does performance improvement correspond only to novelty of ARS use? And (e) are there specific types of questions that are best served by clicker use? These are questions that can be addressed by future studies. For the latter, for example, it would be possible to optimize the number and content of questions as proposed by Preszler *et al*. 24 by restrict questions to those that stimulate interaction and cooperative learning. Optimization of question number, content and revote instances would minimize clicker fatigue, particularly if clicker use became universal throughout a degree program. Instructors certainly will progress in understand the advantages of ARS and how to employ them effectively; nevertheless, it will prove advantageous to engage ARS use with several instruction strategies in order to avoid routine and monotony in teaching 5.

## Conclusions

The introduction of clickers into tutorial teaching helped students to increase exam scores with the effect of lowering the failure rate for students taking the Enzymology course during the second year of undergraduate study at the University of Bordeaux. Our results follow on from positive reports of ARS use in other challenging disciplines, such as physics, mathematics and biology. This is the first report of clicker use for teaching in enzymology. In addition, the novelty of this study relates to the first description of ARS use in small groups to support traditional learning within a large‐group setting. We conclude that clickers used within the question–response framework prepared students better for exams. A very important consideration of this finding is a cost savings that can be attributed to the reduced number of clickers required for small‐group instruction.

## Author contributions

CS‐C was the major contributor to this study and PP helped design it. CS‐C, PP and MAH analyzed and interpreted data and wrote the manuscript.

## Conflict of interest

The authors declare no conflict of interest.
